# Global Existence and Weak-Strong Uniqueness for Chemotaxis Compressible Navier–Stokes Equations Modeling Vascular Network Formation

**DOI:** 10.1007/s00021-023-00840-5

**Published:** 2024-01-18

**Authors:** Xiaokai Huo, Ansgar Jüngel

**Affiliations:** 1 Department of Mathematics, Iowa State University, 411 Morrill Road, Ames, IA 50011-2104 USA; 2 Institute of Analysis and Scientific Computing, Technische Universität Wien, Wiedner Hauptstrasse 8–10, 1040 Wien, Austria

**Keywords:** Compressible Navier–Stokes equations, Chemotaxis force, Global existence of solutions, Weak–strong uniqueness, Relative energy, 35Q30, 35K57, 35K65, 76N05

## Abstract

A model of vascular network formation is analyzed in a bounded domain, consisting of the compressible Navier–Stokes equations for the density of the endothelial cells and their velocity, coupled to a reaction-diffusion equation for the concentration of the chemoattractant, which triggers the migration of the endothelial cells and the blood vessel formation. The coupling of the equations is realized by the chemotaxis force in the momentum balance equation. The global existence of finite energy weak solutions is shown for adiabatic pressure coefficients $\gamma >8/5$. The solutions satisfy a relative energy inequality, which allows for the proof of the weak–strong uniqueness property.

## Introduction

The formation of blood vessels is regulated by chemical signals triggering the movement of endothelial cells. The cells may self-assemble into a vascular network, which is known as vasculogenesis. In this paper, we analyze a mathematical model for the formation of vascular networks, based on mass and momentum balance equations including a chemotaxis force and coupled with a reaction-diffusion equation for the signal concentration. The existence of global weak solutions to the resulting chemotaxis compressible Navier–Stokes equations was proved in [1] for pressures with adiabatic exponent $\gamma >3$. We extend the existence result to the range $\gamma > 8/5$ and prove a weak–strong uniqueness property. The proofs are based on a new relative energy inequality.

The dynamics of the density $\rho (x,t)$ of the endothelial cells, their velocity *v*(*x*, *t*), and the concentration *c*(*x*, *t*) of the chemoattractant (e.g. the vascular endothelial growth factor VEGF-A [17]) is given by the equations

1 $$\begin{aligned}&\partial _t\rho + {\text {div}}(\rho v) = 0, \end{aligned}$$

2 $$\begin{aligned}&\partial _t(\rho v) + {\text {div}}(\rho v\otimes v) + \nabla p(\rho ) = \mu \Delta v + (\lambda +\mu )\nabla {\text {div}}v + \rho \nabla c - \frac{\rho v}{\zeta }, \end{aligned}$$

3 $$\begin{aligned}&\partial _t c = \Delta c - c + \rho \quad \text{ in } \Omega ,\ t>0, \end{aligned}$$

where $\Omega \subset {{\mathbb {R}}}^3$ is a bounded domain, $p(\rho )=\rho ^\gamma$ with the adiabatic exponent $\gamma >1$ is the pressure, the Lamé viscosity constants $\mu$, $\lambda$ satisfy $\mu >0$ and $3\lambda + 2\mu >0$, and $\zeta >0$ is a relaxation constant. We impose the initial and boundary conditions

4 $$\begin{aligned}&\rho (\cdot ,0) = \rho ^0, \quad v(\cdot ,0) = v^0, \quad c(\cdot ,0) = c^0 \quad \text{ in } \Omega , \end{aligned}$$

5 $$\begin{aligned}&v = 0, \quad \nabla c\cdot \nu = 0 \quad \text{ on } \partial \Omega ,\ t>0. \end{aligned}$$

The boundary condition for the velocity *v* is the no-slip condition, and the no-flux boundary condition for *c* means that there is no inflow or outflow of the concentration. The momentum balance equation (2) includes viscous terms as in [1] (suggested in [2, p. 1862]) as well as the chemotaxis force $\rho f_{\textrm{chem}}=-\rho \nabla c$ and the drag force $\rho f_{\textrm{drag}}=-\rho v/\zeta$. The reaction-diffusion equation (3) for the signal concentration models diffusion in the surrounding medium, degradation of the signal in finite time, and the release of the signal produced by the cells. We have set the physical constants in (1)–(3) equal to one, except $\zeta$ to distinguish terms originating from the drag force.

The existence of global finite energy weak solutions to (1)–(5) has been proved in [1] for $\gamma >3$. This restriction comes from the estimation of the chemotaxis force; see Remark 4 on page 10. We extend the existence result to $\gamma >8/5$ by rewriting the force term $\rho \nabla c$ via (3) as $(\partial _t c-\Delta c+c)\nabla c$ and exploiting the properties of the Bogovskii operator. Replacing the parabolic equation (3) for *c* by the elliptic one, we can even allow for $\gamma > 3/2$, which is the condition needed in the existence theory of the compressible Navier–Stokes equations [8]; see Remark 6. This may indicate that our condition $\gamma >8/5$ for system (1)–(3) is not optimal. We discuss this issue further in Remark 5.

The idea of the existence proof in [1] is to derive a priori estimate via the energy-type functional

$$\begin{aligned} {\widetilde{H}}(\rho ,v,c) = \int _\Omega \bigg (\psi (\rho ) + \frac{1}{2}\rho |v|^2 + \frac{1}{2} c^2\bigg )dx, \end{aligned}$$

where $\psi (\rho )=\rho \int _0^\rho s^{-2}p(s)ds=\rho ^\gamma /(\gamma -1)$ can be interpreted as the internal energy. Unfortunately, this functional is not bounded as $t\rightarrow \infty$. Our idea is to use the physical (free) energy,

6 $$\begin{aligned} E(\rho ,v,c) = \int _\Omega \bigg (\psi (\rho ) + \frac{1}{2}\rho |v|^2 + \frac{1}{2}(|\nabla c|^2+c^2) - \rho c\bigg )dx, \end{aligned}$$

which is the sum of the kinetic energy $\frac{1}{2}\int _\Omega \rho |v|^2dx$ and the energy $E(\rho ,0,c)$ of the parabolic–parabolic Keller–Segel model. We show in Sect. 2 (see Lemma 3 on page 6) that

$$\begin{aligned} \frac{dE}{dt}(\rho ,v,c) + \int _\Omega \big (\mu |\nabla v|^2 + (\lambda +\mu )|{\text {div}}v|^2\big )dx + \int _\Omega |\partial _t c|^2 dx \le 0, \end{aligned}$$

providing a bound for $E((\rho ,v,c)(t))$ uniformly in time. Clearly, to infer a priori estimates, we need an upper bound for $\rho c$. This is done by using the inequality

7 $$\begin{aligned} \int _\Omega \rho cdx \le \frac{1}{2}\Vert \psi (\rho )\Vert _{L^1(\Omega )} + \frac{1}{4}\Vert \nabla c\Vert _{L^2(\Omega )}^2 + C_1(\gamma )\Vert c\Vert _{L^1(\Omega )}^{C_2(\gamma )}, \end{aligned}$$

which is due to Sugiyama [18] (see Lemma 9 on page 21), where $C_1(\gamma )>0$ and $C_2(\gamma )>0$ only depend on $\gamma$, and which (7) requires the condition $\gamma >8/5$. The first two terms on the right-hand side of (7) can be absorbed by the energy, while the $L^1(\Omega )$ norm of *c* can be bounded in terms of the initial data $(\rho ^0,c^0)$. This provides a bound for the modified energy-type functional

8 $$\begin{aligned} H(\rho ,v,c) = \frac{1}{2}\int _\Omega \bigg (\psi (\rho ) + \rho |v|^2 + \frac{1}{2}|\nabla c|^2 + c^2\bigg )dx, \end{aligned}$$

namely

$$\begin{aligned} H((\rho ,v,c)(t))&+ \int _0^t\int _\Omega \big (\mu |\nabla v|^2+(\lambda +\mu )|{\text {div}}v|^2\big )dxds \\&+ \int _0^t\int _\Omega |\partial _s c|^2 dxds \le C(\rho ^0,v^0,c^0), \end{aligned}$$

which allows us to prove the global existence of finite energy weak solutions such that $H(\rho ,v,c)$ is finite for all $t>0$. This type of solutions is defined as follows.

### Definition 1

*(Finite energy weak solution)*. The triple $(\rho ,v,c)$ is a *finite energy weak solution* to (1)–(5) if

- they satisfy the regularity
  $$\begin{aligned}&\rho \in L^\infty (0,T;L^\gamma (\Omega )), \quad \rho \ge 0 \text{ in } \Omega ,\ t>0, \\&v\in L^2(0,T;H_0^1(\Omega ;{{\mathbb {R}}}^3)), \quad c\in L^\infty (0,T;H^1(\Omega ))\cap H^1(0,T;L^2(\Omega )); \end{aligned}$$
- equation (1) is satisfied in the sense of renormalized solutions [6, Section 10.18];
- equations (2)–(3) are satisfied in the sense of distributions;
- the energy inequality
  $$\begin{aligned}&E((\rho ,v,c)(t)) + \int _0^t\int _\Omega \big (\mu |\nabla v|^2 + (\lambda +\mu )|{\text {div}}v|^2\big )dxds \\&\quad + \int _0^t\int _\Omega |\partial _s c_n|^2 dxds + \frac{1}{\zeta }\int _0^t\int _\Omega \rho _n|v_n|^2 dxds \le E(\rho ^0,v^0,c^0) \end{aligned}$$
  holds for a.e. $$t\in (0,T)$$.

We introduce for $1<p,q<\infty$ the space $W^{2-2/p,q}_\nu ( \Omega )$ as the completion of the space of functions $w\in C^\infty ({\overline{\Omega }})$ satisfying $\nabla w\cdot \nu =0$ on $\partial \Omega$ in the norm of $W^{2-2/p,q}(\Omega )$. We can now state our first main result.

### Theorem 1

*(Global existence)*. Let $\partial \Omega \in C^2$, $p(\rho )=\rho ^\gamma$ for $\rho \ge 0$ with $\gamma >8/5$. Assume that the initial datum satisfies $\rho ^0\in L^\gamma (\Omega )$ with $\rho ^0\ge 0$, $\rho ^0\not \equiv 0$ in $\Omega$, $\rho ^0|v^0|^2\in L^1(\Omega )$, and $c^0\in W^{2-2/\gamma ,\gamma }_\nu (\Omega )$, $c^0\ge 0$ in $\Omega$. Then there exists a finite energy weak solution $(\rho ,v,c)$ to (1)–(5) in the sense of Definition 1.

The condition on the initial datum $c^0\in W^{2-2/\gamma ,\gamma }_\nu (\Omega )$ can be rephrased in terms of interpolation or Besov spaces. Indeed, the condition is needed to apply the maximal regularity result of Theorem 10, and the regularity on the initial datum can be formulated in such spaces; see [6, Theorem 10.22]. The definition of the pressure can be relaxed to $p\in C^0([0,\infty ))\cap C^2(0,\infty )$, $p(0)=0$, $p'(\rho )>0$ for $\rho >0$, and $\rho ^{1-\gamma }p'(\rho )\rightarrow a>0$ as $\rho \rightarrow \infty$; see [7, (2.1)]. The proof of the theorem is based on the existence theory for the compressible Navier–Stokes equations [8]. More precisely, we add some artificial diffusion and an artificial pressure term, construct Faedo–Galerkin solutions to the approximate problem, prove an approximate energy inequality for these solutions, and pass to the de-regularizing limit. Improved uniform bounds for the cell density in $L^{\gamma +\theta }(\Omega )$ for some $\theta >0$ are derived by testing the mass balance equation with a test function involving the Bogovskii operator. The novel part is the estimate of the chemotaxis force term.

Next, we formulate the weak–strong uniqueness property of the system, meaning that a weak and a strong solution emanating from the same initial data coincide as long as the latter exists.

### Theorem 2

(Weak–strong uniqueness). Let $(\rho ,v,c)$ and $({\bar{\rho }},{{\bar{v}}},{{\bar{c}}})$ be two finite energy weak solutions to (1)–(5) constructed in Theorem 1 with the same initial data. Assume that $({\bar{\rho }},{{\bar{v}}},{{\bar{c}}})$ satisfies the additional regularity

9 $$\begin{aligned} 0<c_p\le {\bar{\rho }}\le C_p, \ |{{\bar{v}}}|\le C_v \text{ a.e. } \text{ in } \Omega \times (0,T), \quad |\nabla {\bar{\rho }}|,\,|\nabla ^2{{\bar{v}}}|\in L^2(0,T;L^q(\Omega )) \end{aligned}$$

for $q>3$ and some constants $c_p,C_p,C_v>0$. Then $\rho ={\bar{\rho }}$, $v={{\bar{v}}}$, and $c={{\bar{c}}}$ in $\Omega \times (0,T)$.

The no-vacuum assumption ${\bar{\rho }}\ge c_p>0$ was also needed in [9] and in related contexts, e.g. for the weak–strong uniqueness property of Maxwell–Stefan systems [11]. The proof of Theorem 2 is based on the relative energy method. The relative energy, associated to the energy functional (6), is given by

$$\begin{aligned} E(\rho ,v,c|r,u,z) = \int _\Omega \bigg (\psi (\rho |r) + \frac{1}{2}\rho |v-u|^2 + \frac{1}{2}\big (|\nabla (c-z)|^2 + (c-z)^2\big ) - (\rho -r)(c-z)\bigg )dx, \end{aligned}$$

where $\psi (\rho |r) = \psi (\rho )-\psi (r)-\psi '(r)(\rho -r)$ is the Bregman distance associated to $\psi$. We show in Lemma 7 on page 11 that

10 $$\begin{aligned}&E((\rho ,v,c)(t)|(r,u,z)(t)) + \int _0^t\int _\Omega \big (\mu |\nabla (v-u|^2 + (\lambda +\mu )|{\text {div}}(v-u)|^2\big )dxds \nonumber \\&\quad + \int _0^t\int _\Omega |\partial _s(c-z)|^2 dxds \le E(\rho ^0,v^0,c^0|r^0,u^0,z^0) + \int _0^t R(\rho ,v,c|r,u,z)ds, \end{aligned}$$

where $(\rho ,v,c)$ is a finite energy weak solution to (1)–(5), (*r*, *u*, *z*) are smooth functions, and the remainder $R(\rho ,v,c|r,u,z)$ is defined in Lemma 7 below. Finite energy weak solutions to the compressible Navier–Stokes equations satisfying (10) have been called *suitable weak solutions* in [9]. It was shown in [7] that finite energy weak solutions in fact always satisfy the relative energy inequality (10) for smooth functions (*r*, *u*, *z*).

Defining the modified relative energy

$$\begin{aligned} H(\rho ,v,c|r,u,z) = \frac{1}{2}\int _\Omega \bigg (\psi (\rho |r) + \rho |v-u|^2 + \frac{1}{2}|\nabla (c-z)|^2 + (c-z)^2\bigg )dx \end{aligned}$$

and giving another weak solution $(r,u,z)=({\bar{\rho }},{\bar{v}},{\bar{c}})$ satisfying the regularity (9), the idea of the proof is to show that

$$\begin{aligned}&R(\rho ,v,c|{\bar{\rho }},{\bar{v}},{\bar{c}}) \le CH(\rho ,v,c|{\bar{\rho }},{\bar{v}},{\bar{c}}) \quad \text{ and } \\&\int _\Omega (\rho -{\bar{\rho }})(c-{\bar{c}})dx \le \frac{1}{2} H(\rho ,v,c|{\bar{\rho }},{\bar{v}},{\bar{c}}) + C(\rho ^0,c^0), \end{aligned}$$

which leads to

$$\begin{aligned} \frac{1}{2} H((\rho ,v,c)(t)|({\bar{\rho }},{\bar{v}},{\bar{c}})(t)) \le E(\rho ^0,v^0,c^0|r^0,u^0,z^0) + C(\rho ^0,c^0) + C\int _0^t H(\rho ,v,c|{\bar{\rho }},{\bar{v}},{\bar{c}})ds \end{aligned}$$

and which implies, by Gronwall’s lemma, that $H((\rho ,v,c)(t)|({\bar{\rho }},{\bar{v}},{\bar{c}})(t))=0$, Consequently $\rho (t)={\bar{\rho }}(t)$, $v(t)={{\bar{v}}}(t)$, and $c(t)={{\bar{c}}}(t)$ for $t>0$.

We finish the introduction by discussing the state of the art. The global existence of finite energy weak solutions to the compressible Navier–Stokes equations with adiabatic exponents $\gamma >3/2$ was shown in [8]. The range of $\gamma$ can be extended to $\gamma >1$ for axisymmetric initial data [12] or for a class of density-dependent viscosity coefficients [14], for instance. Germain [10] proved a relative energy inequality and established the weak–strong uniqueness property for solutions to the compressible Navier–Stokes equations with an integrable spatial density gradient. Feireisl et al. [9] proved the existence of so-called suitable weak solutions satisfying a general relative energy inequality with respect to any sufficiently regular pair of functions and concluded the weak–strong uniqueness property.

Compressible Euler equations with chemotaxis force have been introduced in [17] to describe early stages of vascologenesis. As remarked in [2, Section 3], the fluid equations may also include viscous terms. This leads to chemotaxis compressible Navier–Stokes equations, which have been analyzed in [1] with the pressure function $p(\rho )=\max \{0,\rho -\rho _c\}^\gamma$, where $\gamma >3$ and $\rho _c>0$ is the so-called close-packing density. A viscoelastic mechanical interaction of the cells with the substratum was added to the compressible Euler equations in [20]. Related models are the incompressible Navier–Stokes equations coupled to the chemotaxis Keller–Segel system via the fluid velocity, proposed in [21] and analyzed in, e.g., [22].

The paper is organized as follows. Section 2 is devoted to the proof of Theorem 1. The technical relative energy inequality (10) is proved in Sect. 3. Based on this inequality, Theorem 2 is then shown in Sect. 4. Finally, some auxiliary results are presented in Appendix 4.

## Global Existence of Solutions

In this section, we prove Theorem 1. For this, we proceed as in [8] by constructing an approximate scheme based on a regularized system, deriving uniform energy estimates, and assing to the de-regularization limit. For later use, we note the relations between the pressure $p(\rho )$ and the associated internal energy $\psi (\rho )=\rho \int _0^\rho s^{-2}p(s)ds$:

11 $$\begin{aligned} p(\rho ) = \rho \psi '(\rho )-\psi (\rho ), \quad \nabla p(\rho ) = \rho \nabla \psi '(\rho ) \quad \text{ for } \text{ smooth } \rho . \end{aligned}$$

### Regularized System

We solve first the following regularized system for $\delta >0$, $\varepsilon >0$, and $\beta >4$:

12 $$\begin{aligned}&\partial _t\rho + {\text {div}}(\rho v) = \varepsilon \Delta \rho , \quad \partial _t c = \Delta c - c + \rho , \end{aligned}$$

13 $$\begin{aligned}&\partial _t(\rho v) + {\text {div}}(\rho v\otimes v) + \nabla p(\rho ) + \varepsilon \nabla \rho \cdot \nabla v + \delta \nabla \rho ^\beta \nonumber \\&\quad = \mu \Delta v + (\lambda +\mu )\nabla {\text {div}}v + \rho \nabla c - \frac{\rho v}{\zeta }\quad \text{ in } \Omega ,\ t>0, \end{aligned}$$

subject to the initial and boundary conditions

14 $$\begin{aligned}&\rho (\cdot ,0) = \rho ^0_{\delta }, \quad v(\cdot ,0) = v^0, \quad c(\cdot ,0) = c^0 \quad \text{ in } \Omega , \end{aligned}$$

15 $$\begin{aligned}&\nabla \rho \cdot \nu = 0, \quad v = 0, \quad \nabla c\cdot \nu = 0 \quad \text{ on } \partial \Omega ,\ t>0, \end{aligned}$$

where $\rho ^0_{\delta }$ is a smooth strictly positive function such that $\rho ^0_{\delta }\rightarrow \rho ^0$ strongly in $L^\gamma (\Omega )$. The artificial viscosity term $\varepsilon \Delta \rho$ is balanced by the term $\varepsilon \nabla \rho \cdot \nabla v$ in the momentum equation to control the energy. The artificial pressure term $\delta \nabla \rho ^\beta$ is needed to derive an $L^{\gamma +\theta }(\Omega )$ estimate for the density with $\theta >0$.

The existence of strong solutions to (12)–(15) was shown in [8, Section 2] without the chemotaxis term $\rho \nabla c$. Here, we sketch the proof for the problem including the chemotaxis coupling. As in [8], we use the Faedo–Galerkin method. Let $(\psi _n)$ be a sequence of eigenfunctions of the Laplacian with homogeneous Dirichlet boundary conditions and let $X_n={\text {span}}\{\psi _1,\ldots ,\psi _n\}$. Then, following the proof of [16, Section 7.7] or [5, Chapter 7], we obtain the existence of a unique local strong solution $(\rho _n,v_n,c_n)$ on $(0,T_n)$ such that $v_n\in C^1([0,T_n];X_n)$ and

$$\begin{aligned}&\rho _n,\,\partial _t\rho _n,\,\nabla \rho _n,\,\nabla ^2\rho _n,\,c_n,\,\partial _t c_n,\,\nabla c_n,\,\nabla ^2 c_n \quad \text { are } \text { H}\ddot{{\text {o}}}\text {lder} \text { continuous } \text { on } {\overline{\Omega }}\times [0,T_n], \\ {}&\rho _n(x,t)>0, \quad c_n(x,t)\ge 0\quad \text { for } \text { any } (x,t)\in {\overline{\Omega }}\times [0,T_n]. \end{aligned}$$

To obtain global solutions, i.e. $T=T_n$, we derive an energy inequality for the approximate system.

### Energy Inequality for the Approximate System

An energy-type inequality has been derived in [1, Section 2.2]. Here, we use a different energy functional by including the $H^1(\Omega )$ norm of *c*. We show an inequality for the energies $E(\rho ,v,c)$ and $H(\rho ,v,c)$, defined in (6) and (8), respectively.

#### Lemma 3

Let $(\rho _n,v_n,c_n)$ be a strong solution to (12)–(15) constructed in the previous subsection. Then there exists $C>0$ independent of $(n,\delta ,\varepsilon )$ such that for any $0<t<T_n$,

$$\begin{aligned}&E((\rho _n,v_n,c_n)(t)) + \int _0^t\int _\Omega \big (\mu |\nabla v_n|^2 + (\lambda +\mu )|{\text {div}}v_n|^2\big )dxds + \frac{4\delta \varepsilon }{\beta }\int _0^t\int _\Omega |\nabla \rho _n^{\beta /2}|^2dxds \\&\qquad + \frac{4\varepsilon }{\gamma }\int _0^t\int _\Omega |\nabla \rho _n^{\gamma /2}|^2 dxds + \bigg (1-\frac{\varepsilon }{4}\bigg )\int _0^t\int _\Omega |\partial _s c_n|^2 dx ds + \frac{1}{\zeta }\int _0^t\int _\Omega \rho _n|v_n|^2 dxds \\&\quad \le E(\rho ^0,v^0,v^0) + 2\varepsilon \int _\Omega \rho ^\gamma dx + C\varepsilon , \\&H((\rho _n,v_n,c_n)(t)) + \int _0^t\int _\Omega \big (\mu |\nabla v_n|^2 + (\lambda +\mu )|{\text {div}}v_n|^2\big )dxds \\&\qquad + \frac{4\delta \varepsilon }{\beta }\int _0^t\int _\Omega |\nabla \rho _n^{\beta /2}|^2 dxds + \bigg (1-\frac{\varepsilon }{4}\bigg )\int _\Omega \int _\Omega |\partial _s c_n|^2 dxds + \frac{1}{\zeta }\int _0^t\int _\Omega \rho _n|v_n|^2 dxds \\&\quad \le \big (E(\rho ^0_{\delta },v^0,c^0) + C(\rho ^0,v^0) + C\varepsilon t\big )e^{C\varepsilon t}. \end{aligned}$$

#### Proof

*Step 1: Energy inequality for* *E*. We choose the test function $\psi '(\rho _n)-\frac{1}{2}|v_n|^2+\delta \beta \rho _n^{\beta -1}/(\beta -1)$ in the weak formulation of the first equation in (12) and the test function $v_n$ in the weak formulation of (13). Adding both equations and taking into account (11), some terms cancel, and we arrive after a standard computation at

16 $$\begin{aligned}&\frac{d}{dt}\int _\Omega \bigg (\psi (\rho _n) + \frac{1}{2}\rho _n|v_n|^2 + \frac{\delta }{\beta -1}\rho _n^\beta \bigg )dx + \int _\Omega \big (\mu |\nabla v_n|^2 + (\lambda +\mu )|{\text {div}}v_n|^2\big )dx \nonumber \\&\quad + \frac{1}{\zeta }\int _\Omega \rho _n|v_n|^2 dx + \frac{4\delta \varepsilon }{\beta }\int _\Omega |\nabla \rho _n^{\beta /2}|^2dx + \frac{4\varepsilon }{\gamma }\int _\Omega |\nabla \rho _n^{\gamma /2}|^2 dx = \int _\Omega \rho _n\nabla c_n\cdot v_n dx. \end{aligned}$$

We estimate the right-hand side by integrating by parts and using equation (12) for $\rho _n$:

17 $$\begin{aligned} \int _\Omega \rho _n\nabla c_n\cdot v_n dx&= -\int _\Omega c_n{\text {div}}(\rho _n v_n)dx = \int _\Omega c_n(\partial _t\rho _n-\varepsilon \Delta \rho _n)dx \nonumber \\&= \frac{d}{dt}\int _\Omega \rho _n c_n dx - \int _\Omega \rho _n\partial _t c_n dx - \varepsilon \int _\Omega c_n\Delta \rho _n dx. \end{aligned}$$

Taking into account the second equation in (12), the second term on the right-hand side is written as

$$\begin{aligned} -\int _\Omega \rho _n\partial _t c_n dx&= -\int _\Omega (\partial _t c_n-\Delta c_n+c_n)\partial _t c_ndx \\&= -\int _\Omega |\partial _t c_n|^2 dx - \frac{1}{2}\frac{d}{dt}\int _\Omega (|\nabla c_n|^2 + c_n^2)dx. \end{aligned}$$

Because of $\rho _n c_n\ge 0$, the last term on the right-hand side of (17) becomes

$$\begin{aligned} -\varepsilon \int _\Omega c_n\Delta \rho _n 
dx&= -\varepsilon \int _\Omega \rho _n\Delta c_n dx = -\varepsilon \int _\Omega \rho _n(\partial _t c_n + c_n - \rho _n)dx \\&\le -\varepsilon \int _\Omega \rho _n\partial _t c_n dx + \varepsilon \int _\Omega \rho _n^2 dx \le \frac{\varepsilon }{4}\int _\Omega |\partial _t c_n|^2 dx + 2\varepsilon \int _\Omega \rho _n^2 dx \\&\le \frac{\varepsilon }{4}\int _\Omega |\partial _t c_n|^2 dx + 2\varepsilon \int _\Omega \rho _n^\gamma dx + C(\gamma ,\Omega )\varepsilon , \end{aligned}$$

where the last inequality follows from $\gamma \ge 2$. (We observe that at this point, we can weaken the condition to $\gamma >8/5$ by using the Gagliardo–Nirenberg inequality and the estimate for $\Vert \nabla \rho _n^{\gamma /2}\Vert _{L^2(\Omega )}$ from (16).) We insert these estimates into (17):

$$\begin{aligned} \int _\Omega \rho _n\nabla c_n\cdot v_n dx&\le -\frac{d}{dt}\int _\Omega \bigg (\frac{1}{2}(|\nabla c_n|^2 + c_n^2) - \rho _n c_n\bigg )dx - \int _\Omega |\partial _t c_n|^2 dx \\&\quad + \frac{\varepsilon }{4}\int _\Omega |\partial _t c_n|^2 dx + 2\varepsilon \int _\Omega \rho _n^\gamma dx + C(\gamma ,\Omega )\varepsilon . \end{aligned}$$

Therefore, (16) leads to

18 $$\begin{aligned}&\frac{d}{dt}\int _\Omega \bigg (\frac{1}{2}\rho _n|v_n|^2 + \psi (\rho _n) + \frac{1}{2}(|\nabla c_n|^2 + c_n^2) - \rho _n c_n + \frac{\delta }{\beta -1}\rho _n^\beta \bigg )dx \nonumber \\&\quad + \int _\Omega \big (\mu |\nabla v_n|^2 + (\lambda +\mu )|{\text {div}}v_n|^2\big )dx + \frac{4\delta \varepsilon }{\beta }\int _\Omega |\nabla \rho _n^{\beta /2}|^2dx + \frac{4\varepsilon }{\gamma }\int _\Omega |\nabla \rho _n^{\gamma /2}|^2 dx \nonumber \\&\quad + \bigg (1-\frac{\varepsilon }{4}\bigg )|\partial _t c_n|^2 dx + \frac{1}{\zeta }\int _\Omega \rho _n|v_n|^2 dx \le 2\varepsilon \int _\Omega \rho _n^\gamma dx + C\varepsilon , \end{aligned}$$

where $C>0$ only depends on $\gamma$ and $\text{ meas }(\Omega )$ but is independent of *n*, $\delta$, and $\varepsilon$. This proves the inequality for $E(\rho _n,v_n,c_n)$.

*Step 2: Energy inequality for* *H*. We need to estimate $\int _\Omega \rho _n c_n dx$ in $E(\rho _n,v_n,c_n)$. By Lemma 9 in Appendix 4, applied to $m=\gamma$, $\kappa =1/(2(\gamma -1))$, and $\xi =1/4$,

19 $$\begin{aligned} \int _\Omega \rho _n c_n dx \le \frac{1}{2(\gamma -1)}\Vert \rho _n\Vert _{L^\gamma (\Omega )}^\gamma + \frac{1}{4}\Vert \nabla c_n\Vert _{L^2(\Omega )}^2 + C_1(\gamma )\Vert c_n\Vert _{L^1(\Omega )}^{C_2(\gamma )}. \end{aligned}$$

Equation (12) implies that the mass is conserved, $\Vert \rho _n(t)\Vert _{L^1(\Omega )} = \Vert \rho ^0_{\delta }\Vert _{L^1(\Omega )}$ for $0<t<T_n$. Furthermore, by the second equation in (12),

$$\begin{aligned} \frac{d}{dt}\int _\Omega c_n dx = \int _\Omega \rho _n dx - \int _\Omega c_n dx. \end{aligned}$$

This is an ordinary differential equation for $t\mapsto \Vert c_n(t)\Vert _{L^1(\Omega )}$, and a comparison principle as well as the nonnegativity of $c_n$ imply that

$$\begin{aligned} \Vert c_n(t)\Vert _{L^1(\Omega )} = \int _\Omega c_n dx \le \max \bigg \{\int _\Omega c^0dx, \int _\Omega \rho ^0_{\delta }dx\bigg \} \le C, \end{aligned}$$

where $C>0$ is independent of $\delta$. Thus, we conclude from (19) and $\rho _n^\gamma /(2(\gamma -1)) = \frac{1}{2}\psi (\rho _n)$ that

$$\begin{aligned} \int _\Omega \rho _n c_n dx \le \frac{1}{2}\int _\Omega \psi (\rho _n) dx + \frac{1}{4}\Vert \nabla c_n\Vert _{L^2(\Omega )}^2 + C(\rho ^0,c^0). \end{aligned}$$

It follows from the definitions of $E(\rho _n,v_n,c_n)$ and $H(\rho _n,v_n,c_n)$ that

$$\begin{aligned} E(\rho _n,v_n,c_n)&\ge \int _\Omega \bigg (\frac{1}{2}\psi (\rho _n) + \frac{1}{2}\rho _n|v_n|^2 + \frac{1}{4}|\nabla c_n|^2 + \frac{1}{2} c_n^2\bigg )dx - C(\rho ^0,c^0) \\&= H(\rho _n,v_n,c_n) - C(\rho ^0,c^0). \end{aligned}$$

We insert these estimates in (18) and integrate over (0, *t*) for $0<t<T_n$:

$$\begin{aligned}&H((\rho _n,v_n,c_n)(t)) + \int _0^t\int _\Omega \big (\mu |\nabla v_n|^2 + (\lambda +\mu )|{\text {div}}v_n|^2\big )dxds + \frac{4\delta \varepsilon }{\beta }\int _0^t\int _\Omega |\nabla \rho _n^{\beta /2}|^2 dxds \\&\qquad + \frac{4\varepsilon }{\gamma }\int _0^t\int _\Omega |\nabla \rho _n^{\gamma /2}|^2 dxds + \bigg (1-\frac{\varepsilon }{4}\bigg )\int _0^t\int _\Omega |\partial _s c_n|^2 dxds + \frac{1}{\zeta }\int _0^t\int _\Omega \rho _n|v_n|^2 dxds \\&\quad \le E(\rho ^0_{\delta },v^0,c^0) + C\varepsilon \int _0^t\int _\Omega H(\rho _n,v_n,c_n)dxds + C(\rho ^0,c^0) + C\varepsilon t, \end{aligned}$$

where we used $\int _\Omega \rho _n^\gamma dx\le CH(\rho _n,v_n,c_n)$. An application of Gronwall’s lemma finishes the proof. $\square$

Lemma 3 allows us to conclude as in [8, Section 2.3] that $T=T_n$. Moreover, it yields the following estimates uniform in $(n,\delta ,\varepsilon )$:

20 $$\begin{aligned} \begin{aligned} (\rho _n)&\quad \text{ is } \text{ uniformly } \text{ bounded } \text{ in } L^\infty (0,T;L^\gamma (\Omega )), \\ (\sqrt{\rho _n}v_n)&\quad \text{ is } \text{ uniformly } \text{ bounded } \text{ in } L^\infty (0,T;L^2(\Omega ;{{\mathbb {R}}}^3)), \\ (\nabla v_n)&\quad \text{ is } \text{ uniformly } \text{ bounded } \text{ in } L^2(0,T;L^2(\Omega ;{{\mathbb {R}}}^{3\times 3})), \\ (c_n)&\quad \text{ is } \text{ uniformly } \text{ bounded } \text{ in } L^\infty (0,T;H^1(\Omega ))\cap H^1(0,T;L^2(\Omega )). \end{aligned} \end{aligned}$$

### Limit $(n,\delta ,\varepsilon )\rightarrow (\infty ,0,0)$

The limit $n\rightarrow \infty$ can be performed as in [1, Section 2.3] via the Aubin–Lions compactness lemma. This gives a solution $(\rho _\delta ,v_\delta ,c_\delta )$ to (12)–(15). It satisfies the energy inequalities in Lemma 3. In particular, we conclude a uniform bound for $\rho _\delta$ in $L^\infty (0,T;L^\gamma (\Omega ))$. By the existence theory for the compressible Navier–Stokes equations, we can pass to the limit $(\delta ,\varepsilon )\rightarrow 0$; see, e.g., [5, 16]. Indeed, to derive a uniform estimate for the mass density in $L^{\gamma +\theta }(\Omega \times (0,T))$ for some $\theta >0$, we need to use the test function

$$\begin{aligned} \phi _B = {\mathcal {B}}\bigg (\rho _\delta ^\theta - \frac{1}{|\Omega |} \int _\Omega \rho _\delta ^\theta dx\bigg ), \end{aligned}$$

in the weak formulation of the approximate momentum equation, where ${\mathcal {B}}$ is the Bogovskii operator [16, Section 3.3.1.2]. Compared to the compressible Navier–Stokes equations, the momentum equation includes the chemotaxis term $\rho _\delta \nabla c_\delta$, which needs to be estimated. This means that we need a bound for

21 $$\begin{aligned} I = \int _0^T\int _\Omega \rho _\delta \nabla c_\delta \cdot \phi _B dxdt. \end{aligned}$$

Using the second equation in (12),

$$\begin{aligned} \rho _\delta \nabla c_\delta = (\partial _t c_\delta - \Delta c_\delta + c_\delta )\nabla c_\delta = (\partial _t c_\delta + c_\delta )\nabla c_\delta - {\text {div}}(\nabla c_\delta \otimes \nabla c_\delta ) + \frac{1}{2}\nabla |\nabla c_\delta |^2, \end{aligned}$$

we can write $I=I_1+\cdots +I_4$, where

$$\begin{aligned} I_1&= \int _0^T\int _\Omega \partial _t c_\delta \nabla c_\delta \cdot \phi _B dxdt,&I_2&= \int _0^T\int _\Omega c_\delta \nabla c_\delta \cdot \phi _B dxdt, \\ I_3&= \int _0^T\int _\Omega \nabla c_\delta \otimes \nabla c_\delta :\nabla \phi _B dxdt,&I_4&= -\frac{1}{2}\int _0^T\int _\Omega |\nabla c_\delta |^2 {\text {div}}\phi _B dxdt. \end{aligned}$$

We start with the term $I_1$. First, let $\gamma >2$. By parabolic regularity theory (see Theorem 10 in the Appendix with $p=q=\gamma$), the continuous embedding $W^{2-2/\gamma ,\gamma }(\Omega )\hookrightarrow W^{1,\gamma }(\Omega )$ and the second equation in (12) yield

22 $$\begin{aligned} \Vert \nabla c_\delta \Vert _{L^2(0,T;L^\gamma (\Omega ))}&\le C\Vert c_\delta \Vert _{L^2(0,T;W^{2-2/\gamma ,\gamma }(\Omega ))} \nonumber \\&\le C\big (\Vert \rho _\delta \Vert _{L^\infty (0,T;L^\gamma (\Omega ))} + \Vert c^0\Vert _{W^{2-2/\gamma ,\gamma }(\Omega )}\big ) \le C. \end{aligned}$$

Hence, using Hölder’s inequality, the assumption $\gamma >2$, and the previous estimates as well as the uniform estimates from the energy inequality,

$$\begin{aligned} I_1 \le \Vert \partial _t c_\delta \Vert _{L^2(0,T;L^2(\Omega ))} \Vert \nabla c_\delta \Vert _{L^2(0,T;L^\gamma (\Omega ))} \Vert \phi _B\Vert _{L^\infty (0,T;L^{2\gamma /(\gamma -2)}(\Omega ))} \le C\Vert \phi _B\Vert _{L^\infty (0,T;W^{1,r}(\Omega ))}, \end{aligned}$$

where $r=6\gamma /(5\gamma -6)$ is such that $W^{1,r}(\Omega )\hookrightarrow L^{2\gamma /(\gamma -2)}(\Omega )$. We deduce from the boundedness of ${\mathcal {B}}:L_0^r(\Omega )\rightarrow W_0^{1,r}(\Omega )$ for $1<r<\infty$, where $L_0^r(\Omega )$ is the space of all $L^r(\Omega )$ functions *u* satisfying $\int _\Omega udx=0$, that

$$\begin{aligned} I_1&\le C\bigg \Vert \rho _\delta 
^\theta - \frac{1}{|\Omega |} \int _\Omega \rho _\delta ^\theta dx\bigg \Vert _{L^\infty (0,T;L^r(\Omega ))} \le C\Vert \rho _\delta ^\theta \Vert _{L^\infty (0,T;L^r(\Omega ))} \\&\le C\Vert \rho _\delta \Vert _{L^\infty (0,T;L^{r\theta }(\Omega ))}^\theta \le C\Vert \rho _\delta \Vert _{L^\infty (0,T;L^\gamma (\Omega ))}^\theta \le C. \end{aligned}$$

The last but one step follows if $r\theta \le \gamma$, which requires the choice $0<\theta \le 5\gamma /6-1$, and the last step is a consequence of the energy inequality.

Next, let $3/2<\gamma \le 2$. We apply Theorem 10 with $p=2$, $q=\gamma$ to find that

$$\begin{aligned} \Vert c_\delta \Vert _{L^2(0,T;W^{2,\gamma }(\Omega ))} + \Vert \partial _t c_\delta \Vert _{L^2(0,T;L^\gamma (\Omega ))} \le C\big (\Vert \rho _\delta \Vert _{L^2(0,T;L^\gamma (\Omega ))} + \Vert c^0\Vert _{W^{1,\gamma }(\Omega )}\big ) \le C. \end{aligned}$$

Hence, we deduce from the continuous embedding $W^{2,\gamma }(\Omega )\hookrightarrow W^{1,3\gamma /(3-\gamma )}(\Omega )$ that

$$\begin{aligned} I_1&\le \Vert \partial _t c_\delta \Vert _{L^2(0,T;L^\gamma (\Omega ))} \Vert \nabla c_\delta \Vert _{L^2(0,T;L^{3\gamma /(3-\gamma )}(\Omega ))} \Vert \phi _B\Vert _{L^\infty (0,T;L^{3\gamma /(4\gamma -6)}(\Omega ))} \\&\le C\Vert \partial _t c_\delta \Vert _{L^2(0,T;L^\gamma (\Omega ))} \Vert c_\delta \Vert _{L^2(0,T;W^{2,\gamma }(\Omega ))} \Vert \phi _B\Vert _{L^\infty (0,T;W^{1,r}(\Omega ))}, \end{aligned}$$

where now $r=3\gamma /(5\gamma -6)$. We choose $\theta >0$ such that $r\theta \le \gamma$, which is equivalent to $\theta \le 5\gamma /3-2$, and we can choose $\theta >0$ satisfying this inequality. Then, arguing as in the case $\gamma \ge 2$,

$$\begin{aligned} I_1 \le C\Vert \rho _\delta \Vert _{L^\infty (0,T;L^{r\theta }(\Omega ))}^\theta \le C\Vert \rho _\delta \Vert _{L^\infty (0,T;L^\gamma (\Omega ))}^\theta \le C. \end{aligned}$$

For the term $I_3$, we consider again first the case $\gamma >2$:

$$\begin{aligned} I_3 \le \Vert \nabla c_\delta \Vert _{L^2(0,T;L^\gamma (\Omega ))}^2 \Vert \nabla \phi _B\Vert _{L^\infty (0,T;L^r(\Omega ))} \le C\Vert \rho _\delta \Vert _{L^\infty (0,T;L^{r\theta }(\Omega ))}^\theta \le C, \end{aligned}$$

where $r=\gamma /(\gamma -2)$, and the last inequality follows if $r\theta \le \gamma$, which is equivalent to $\theta \le \gamma -2$. If $3/2<\gamma \le 2$, we proceed similarly as for $I_1$:

$$\begin{aligned} I_3 \le \Vert \nabla c_\delta \Vert _{L^2(0,T;L^{3\gamma /(3-\gamma )}(\Omega ))}^2 \Vert \nabla \phi _B\Vert _{L^\infty (0,T;L^r(\Omega ))} \le C\Vert \rho _\delta \Vert _{L^\infty (0,T;L^{r\theta }(\Omega ))}^\theta \le C, \end{aligned}$$

where $r=\gamma /(2\gamma -3)$ and we need $r\theta \le \gamma$ or, equivalently, $\theta \le 2\gamma -3$.

The term $I_2$ is estimated in a similar way as $I_1$, and $I_4$ can be bounded as $I_3$. This shows that *I* is bounded and provides a uniform estimate for $\rho _\delta$ in $L^{\gamma +\theta }(\Omega \times (0,T))$. Now we can proceed as in [16, Section 7.3] to prove the strong convergence of the pressure.

#### Remark 4

(On the condition on $\gamma$ in [1]) Aïssa and Alexandre have estimated the term *I*, defined in (21), in a different way. They used the test function $\psi _B={\mathcal {B}}(\rho _\delta - |\Omega |^{-1} \int _\Omega \rho _\delta dx)$:

$$\begin{aligned} I&\le \Vert \rho _\delta \Vert _{L^2(\Omega \times (0,T))}\Vert \nabla c_\delta \Vert _{L^2(\Omega \times (0,T))} \Vert \psi _B\Vert _{L^\infty (0,T;L^\infty (\Omega ))} \\&\le \Vert \rho _\delta \Vert _{L^2(\Omega \times (0,T))}\Vert \nabla c_\delta \Vert _{L^2(\Omega \times (0,T))} \Vert \rho _\delta \Vert _{L^\infty (0,T;L^r(\Omega ))}, \end{aligned}$$

which is a consequence of the estimate $\Vert {\mathcal {B}}(f)\Vert _{L^\infty (\Omega )} \le C\Vert {\mathcal {B}}(f)\Vert _{W^{1,r}(\Omega )}\le C\Vert f\Vert _{L^r(\Omega )}$ choosing $r>d=3$. Thus, the technique of [1] only works for $\gamma >3$. $\square$

#### Remark 5

(On the condition $\gamma >8/5$) This restriction is needed to estimate the integral $\int _\Omega \rho _n c_n dx$ by means of Lemma 9. The idea is to obtain “small” terms that can be absorbed by the left-hand side of the energy inequality (18) and terms that can be controlled (the $L^1(\Omega )$ norm of $c_n$). By the Hölder and Gagliardo–Nirenberg inequalities, we may estimate in a different way:

$$\begin{aligned} \int _\Omega \rho _n c_n dx \le \Vert \rho _n\Vert _{L^\gamma (\Omega )} \Vert c_n\Vert _{L^{\gamma /(\gamma -1)}(\Omega )} \le C\Vert \rho _n\Vert _{L^\gamma (\Omega )}\Vert c_n\Vert _{W^{2,\gamma }(\Omega )}^\theta \Vert c_n\Vert _{L^1(\Omega )}^{1-\theta }, \end{aligned}$$

where $\theta =3/(5\gamma -3)\in (0,1)$ (which requires that $\gamma >6/5$). It follows from the maximal regularity result of Theorem 10 that

$$\begin{aligned} \int _\Omega \rho _n c_n dx \le C\Vert \rho _n\Vert _{L^\gamma (\Omega )} (\Vert \rho _n\Vert _{L^\gamma (\Omega )}+1)^\theta , \end{aligned}$$

where $C>0$ depends on $\Vert c^0\Vert _{L^1(\Omega )}$. We can conclude if $1+\theta <\gamma$, which is equivalent to $\gamma >8/5$. Thus, even taking into account maximal regularity does not improve the range for $\gamma$.

#### Remark 6

(Improving the condition on $\gamma$) When the dynamics of the chemical concentration is much faster than that one of the cell density, we can neglect the time derivative of the concentration in (3), and $c_\delta$ solves $0=\Delta c_\delta -c_\delta +\rho _\delta$ in $\Omega$. In this situation, we are able to weaken the condition on $\gamma$ to $\gamma >3/2$. Indeed, estimate (22) still holds for the elliptic problem. The embedding $W^{1,\gamma }(\Omega )\hookrightarrow L^{3\gamma /(3-\gamma )}(\Omega )$ for $\gamma <3$ then shows that

$$\begin{aligned} \Vert \nabla c_\delta \Vert _{L^\infty (0,T;L^{3\gamma /(3-\gamma )}(\Omega ))} \le C\Vert c_\delta \Vert _{L^\infty (0,T;W^{2,\gamma }(\Omega ))} \le C\Vert \rho _\delta \Vert _{L^\infty (0,T;L^\gamma (\Omega ))} \le C. \end{aligned}$$

Hence, using Hölder’s inequality, we estimate

$$\begin{aligned} I_3&\le \Vert \nabla c_\delta \Vert ^2_{L^\infty (0,T;L^{3\gamma /(3-\gamma )}(\Omega ))} \Vert \nabla \phi _B\Vert _{L^\infty (0,T;L^{3\gamma /(5\gamma -6)}(\Omega ))} \\&\le C\Vert \rho _\delta \Vert _{L^\infty (0,T;L^\gamma (\Omega ))}^2 \Vert \rho _\delta ^\theta \Vert _{L^\infty (0,T;L^{3\gamma /(5\gamma -6)}(\Omega ))} \le C+C\Vert \rho _\delta \Vert _{L^\infty (0,T;L^\gamma (\Omega ))}^{2+\theta } \le C, \end{aligned}$$

provided that $0<\theta < (5\gamma -6)/3$. The terms $I_2$ and $I_4$ are estimated in a similar way and $I_1=0$, thus proving that *I* is bounded. This yields a uniform estimate for $\rho _\delta$ in $L^{\gamma +\theta }(\Omega \times (0,T))$ for $\gamma >3/2$ according to the theory of the compressible Navier–Stokes equations. $\square$

## Relative Energy Inequality

We show a relative energy inequality for smooth functions.

### Lemma 7

*(Relative energy inequality)*. Let $(\rho ,v,c)$ be a smooth solution to (1)–(5) and let (*r*, *u*, *z*) be smooth functions satisfying $r>0$ in ${\overline{\Omega }}\times [0,T]$ and $u=0$ on $\partial \Omega$. Then the relative energy inequality (10) holds for $0<t<T$ with

$$\begin{aligned} R(\rho ,v,c|r,u,z)&= -\int _\Omega p(\rho |r){\text {div}}u dx - \int _\Omega \psi ''(r)(\rho -r)g dx - \int _\Omega h\partial _t(c-z)dx \\&\quad - \int _\Omega \nabla (c-z)\cdot ((\rho -r)u)dx + \int _\Omega (c-z)g dx \\&\quad - \int _\Omega \rho (v-u)\otimes (v-u):\nabla u dx - \frac{1}{\zeta }\int _\Omega \rho |v-u|^2 dx \\&\quad - \int _\Omega \bigg (\frac{\rho -r}{r}\big (\mu \Delta u + (\lambda +\mu )\nabla {\text {div}}u\big ) + \rho f\bigg )\cdot (v-u)dx, \end{aligned}$$

where

23 $$\begin{aligned}&f = \partial _t u + u\cdot \nabla u + \frac{1}{r}\nabla p(r) - \frac{1}{r}\big (\mu \Delta u + (\lambda +\mu )\nabla {\text {div}}u\big ) - \nabla z + \frac{u}{\zeta }, \end{aligned}$$

24 $$\begin{aligned}&g = \partial _t r + {\text {div}}(ru), \quad h = \partial _t z - \Delta z + z - r. \end{aligned}$$

We prove in Sect. 4 that the relative energy inequality (10) holds for finite energy weak solutions $(\rho ,v,c)$ and $({\bar{\rho }},{\bar{v}},{\bar{c}})$, where $({\bar{\rho }},{\bar{v}})$ satisfies (9). The proof of (10) follows the lines of [9, Section 3.2], but some steps are different due to the additional chemotaxis force. For this reason, and for the convenience of the reader, we present a full proof.

### Proof

Let $$(r_m,u_m,z_m)_{m\in {{\mathbb {N}}}}$$ be smooth functions satisfying $$r_m>0$$ in $${\overline{\Omega }}\times [0,T]$$, $$v_m\in C^1([0,T];$$ $$X_m)$$, and $$v_m=0$$ on $$\partial \Omega $$ such that $$(r_m,u_m,z_m)\rightarrow (r,z,u)$$ as $$m\rightarrow \infty $$ in a sense made precise in Step 3 below. Here, $$X_m$$ is the Faedo–Galerkin space defined in Sect. 2.1. We introduce

25 $$\begin{aligned} f_m&= \partial _t u_m + u_m\cdot \nabla u_m + \frac{1}{r_m}\nabla p(r_m) - \frac{1}{r_m}\big (\mu \Delta u_m + (\lambda +\mu )\nabla {\text {div}}u_m\big ) \nonumber \\&\quad - \nabla z_m + \frac{u_m}{\zeta }, \end{aligned}$$

26 $$\begin{aligned} g_m&= \partial _t r_m + {\text {div}}(r_mu_m), \quad h_m = \partial _t z_m - \Delta z_m + z_m - r_m. \end{aligned}$$

Then $(f_m,g_m,h_m)\rightarrow (f,g,h)$ as $m\rightarrow \infty$ in the sense of distributions, where (*f*, *g*, *h*) is defined in (23)–(24). Finally, let $(\rho _n,v_n,c_n)$ be a Galerkin solution to (12)–(15). We compute in the following the approximate relative energy inequality.

*Step 1: Time derivative of the relative kinetic energy.* We derive an equation for the time evolution of the relative kinetic energy $\frac{1}{2}\int _\Omega \rho _n|v_n-u_m|^2 dx$. It follows from the approximative mass balance equation (12) that

27 $$\begin{aligned}&\frac{1}{2}\frac{d}{dt}\big (\rho _n|v_n-u_m|^2\big ) = -\frac{1}{2}\big ({\text {div}}(\rho _n v_n) - \varepsilon \Delta \rho _n\big )|v_n-u_m|^2 + \rho _n\partial _t(v_n-u_m)\cdot (v_n-u_m) \nonumber \\&\quad = -\frac{1}{2}{\text {div}}\big (\rho _n v_n|v_n-u_m|^2\big ) + \rho _n v_n\cdot \nabla (v_n-u_m)\cdot (v_n-u_m) \nonumber \\&\qquad + \rho _n\partial _t(v_n-u_m)\cdot (v_n-u_m) + \frac{\varepsilon }{2}\Delta \rho _n|v_n-u_m|^2. \end{aligned}$$

Since $\rho _n(\partial _t v_n+v_n\cdot \nabla v_n)=\partial _t(\rho _n v_n)+{\text {div}}(\rho _n v_n\otimes v_n) - \varepsilon \Delta \rho _n v_n$, the second and third terms on the right-hand side are written as

$$\begin{aligned}&\rho _n v_n\cdot \nabla (v_n-u_m)\cdot (v_n-u_m) + \rho _n\partial _t(v_n-u_m)\cdot (v_n-u_m) \\&\quad = \rho _n(\partial _t v_n+v_n\cdot \nabla v_n)\cdot (v_n-u_m) - \rho _n(\partial _t u_m + v_n\cdot \nabla u_m)\cdot (v_n-u_m) \\&\quad = \big (\partial _t(\rho _n v_n) + {\text {div}}(\rho _n v_n\otimes v_n)\big )\cdot (v_n-u_m) - \varepsilon \Delta \rho _n v_n\cdot (v_n-u_m) \\&\qquad - \rho _n(\partial _t u_m+u_m\cdot \nabla u_m)\cdot (v_n-u_m) - \rho _n(v_n-u_m)\cdot \nabla u_m\cdot (v_n-u_m). \end{aligned}$$

We insert this expression into (27), integrate over $\Omega$, and replace $\partial _t(\rho _n v_n)+{\text {div}}(\rho _n v_n\otimes v_n)$ by the momentum equation (13):

28 $$\begin{aligned}&\frac{1}{2}\frac{d}{dt}\int _\Omega \rho _n|v_n-u_m|^2dx = \frac{\varepsilon }{2}\int _\Omega \Delta \rho _n|v_n-u_m|^2 dx - \int _\Omega \nabla (p(\rho _n)+\delta \rho _n^\beta )\cdot (v_n-u_m)dx \nonumber \\&\quad - \int _\Omega \big (\mu \nabla v_n\cdot \nabla (v_n-u_m) + (\lambda +\mu ){\text {div}}v_n{\text {div}}(v_n-u_m)\big )dx \nonumber \\&\quad - \varepsilon \int _\Omega \nabla \rho _n\cdot \nabla v_n\cdot (v_n-u_m)dx + \int _\Omega \rho _n\nabla c_n\cdot (v_n-u_m)dx \nonumber \\&\quad - \frac{1}{\zeta }\int _\Omega \rho _n v_n\cdot (v_n-u_m)dx - \varepsilon \int _\Omega \Delta \rho _n v_n\cdot (v_n-u_m)dx \nonumber \\&\quad - \int _\Omega \rho _n(\partial _t u_m+u_m\cdot \nabla u_m)\cdot (v_n-u_m)dx - \int _\Omega \rho _n(v_n-u_m)\cdot \nabla u_m\cdot (v_n-u_m)dx. \end{aligned}$$

We wish to reformulate the last but one term in the previous equality. For this, we add and subtract $r_m$ and replace $r_m(\partial _t u_m+u_m\cdot \nabla u_m)$ by (25):

$$\begin{aligned}&-\int _\Omega \rho _n(\partial _t u_m+u_m\cdot \nabla u_m)\cdot (v_n-u_m)dx \\&\quad = -\int _\Omega \bigg (1 + \frac{\rho _n-r_m}{r_m}\bigg )\big (r_m(\partial _t u_m+ u_m\cdot \nabla u_m) \big )\cdot (v_n-u_m)dx \\&\quad = \int _\Omega \bigg (1 + \frac{\rho _n-r_m}{r_m}\bigg )\bigg (\nabla p(r_m) - r_m\nabla z_m + \frac{r_mu_m}{\zeta } - r_mf_m\bigg )\cdot (v_n-u_m)dx \\&\qquad - \int _\Omega \bigg (1 + \frac{\rho _n-r_m}{r_m}\bigg )\big (\mu \Delta u_m + (\lambda +\mu )\nabla {\text {div}}u_m\big )\cdot (v_n-u_m)dx. \end{aligned}$$

Then, after a computation, (28) becomes

29 $$\begin{aligned}&\frac{1}{2}\frac{d}{dt}\int _\Omega \rho _n|v_n-u_m|^2dx = -\int _\Omega \bigg (\nabla p(\rho _n) - \frac{\rho _n}{r_m}\nabla p(r_m)\bigg )\cdot (v_n-u_m)dx \nonumber \\&\quad - \delta \int _\Omega \nabla \rho _n^\beta \cdot (v_n-u_m)dx + \varepsilon \int _\Omega \nabla \rho _n\cdot \nabla u_m\cdot (v_n-u_m)dx \nonumber \\&\quad - \int _\Omega \big (\mu |\nabla (v_n-u_m)|^2 + (\lambda +\mu )|{\text {div}}(u_n-u_m)|^2 \big )dx \nonumber \\&\quad - \int _\Omega \rho _n(v_n-u_m)\otimes (v_n-u_m):\nabla u_m dx + \int _\Omega \rho _n\nabla (c_n-z_m)\cdot (v_n-u_m)dx \nonumber \\&\quad - \frac{1}{\zeta }\int _\Omega \rho _n|v_n-u_m|^2 dx - \int _\Omega \rho _n f_m\cdot (v_n-u_m)dx \nonumber \\&\quad - \int _\Omega \frac{\rho _n-r_m}{r_m}\big (\mu \Delta u_m + (\lambda +\mu ) \nabla {\text {div}}u_m\big )\cdot (v_n-u_m)dx. \end{aligned}$$

We rewrite the first, second, and sixth terms on the right-hand side of (28).

*Step 2a: Reformulation of the pressure term.* Observing that $p'(z)=z\psi ''(z)$ for $z\ge 0$ (see (11)) and that $\rho _m-r_m$ satisfies

$$\begin{aligned} \partial _t(\rho _n-r_m) + {\text {div}}\big ((\rho _n-u_m)u_m + \rho _n(v_n-u_m)\big ) = \varepsilon \Delta \rho _n - g_m, \end{aligned}$$

we can write the first term on the right-hand side of (29) as

30 $$\begin{aligned}&-\int _\Omega \bigg (\nabla p(\rho _n) - \frac{\rho _n}{r_m}\nabla p(r_m)\bigg )\cdot (v_n-u_m)dx = \int _\Omega \rho _n\nabla \big (\psi '(\rho _n)-\psi '(r_m)\big )\cdot (v_n-u_m)dx \nonumber \\&\quad = -\int _\Omega \big (\psi '(\rho _n)-\psi '(r_m)\big ){\text {div}}\big (\rho _n(v_n-u_m)\big )dx \nonumber \\&\quad = -\int _\Omega \big (\psi '(\rho _n)-\psi '(r_m)\big )\partial _t(\rho _n-r_m)dx \nonumber \\&\qquad - \int _\Omega \big (\psi '(\rho _n)-\psi '(r_m)\big ){\text {div}}((\rho _n-r_m)u_m)dx \nonumber \\&\qquad + \int _\Omega \big (\psi '(\rho _n)-\psi '(r_m)\big )(\varepsilon \Delta \rho _n-g_m)dx. \end{aligned}$$

Taking into account that the evolution of the relative internal energy is given by

$$\begin{aligned} \partial _t\psi (\rho _n|r_m)&= \partial _t\big (\psi (\rho _n)-\psi (r_m)-\psi '(r_m)(\rho _n-r_m)\big ) \\&= \big (\psi '(\rho _n)-\psi '(r_m)\big )\partial _t\rho _n - \psi ''(r_m)\partial _t r_m(\rho _n-r_m), \end{aligned}$$

the first term on the right-hand side of (30) is reformulated as

$$\begin{aligned}&-\int _\Omega \big (\psi '(\rho _n)-\psi '(r_m)\big )\partial _t(\rho _n-r_m)dx \\&\quad = -\int _\Omega \big (\psi '(\rho _n)-\psi '(r_m)\big )\partial _t\rho _n dx + \int _\Omega \big (\psi '(\rho _n)-\psi '(r_m)\big )\partial _t r_m dx \\&\quad = -\int _\Omega \bigg (\frac{d}{dt}\psi (\rho _n|r_m) + \psi ''(r_m)\partial _t r_m(\rho _n-r_m)\bigg )dx + \int _\Omega \big (\psi '(\rho _n)-\psi '(r_m)\big )\partial _t r_m dx \\&\quad = -\frac{d}{dt}\int _\Omega \psi (\rho _n|r_m)dx + \int _\Omega \big (\psi '(\rho _n)-\psi '(r_m)-\psi ''(r_m)(\rho _n-r_m)\big ) (g_m-{\text {div}}(r_mu_m))dx, \end{aligned}$$

where we used definition (26) of $g_m$ in the last step. Integrating by parts to get rid of the divergence, inserting the corresponding expression into (30), and observing that the integral over $(\psi '(\rho _n)-\psi '(r_m))g_m$ cancels with the corresponding expression in (30), we find that

$$\begin{aligned}&-\int _\Omega \bigg (\nabla p(\rho _n) - \frac{\rho _n}{r_m}\nabla p(r_m)\bigg )\cdot (v_n-u_m)dx = -\frac{d}{dt}\int _\Omega \psi (\rho _n|r_m)dx \\&\quad + \int _\Omega \big \{\nabla \big (\psi '(\rho _n)-\psi '(r_m)\big )\cdot (\rho _n u_m) - \nabla \big (\psi ''(r_m)(\rho _n-r_m)\big )\cdot (r_mu_m)\big \}dx \\&\quad + \varepsilon \int _\Omega \big (\psi '(\rho _n)-\psi '(r_m)\big )\Delta \rho _n dx - \int _\Omega \psi ''(r_m)(\rho _n-r_m)g_m dx. \end{aligned}$$

We claim that the second term on the right-hand side can be formulated in terms of the relative pressure $p(\rho _n|r_m) = p(\rho _n)-p(r_m)-p'(r_m)(\rho _n-r_m)$. It follows from (11) that $\nabla p(\rho _n)=\rho _n\nabla \psi '(\rho _n)$, $\nabla p'(r_m) = \nabla \psi '(r_m)+r_m\nabla \psi ''(r_m)$ and hence,

$$\begin{aligned} \nabla p(\rho _n|r_m)&= \rho _n\nabla \psi '(\rho _n) - r_m\nabla \psi '(r_m) - \nabla \big (r_m\psi ''(r_m)(\rho _n-r_m)\big ) \\&= \rho _n\nabla \big (\psi '(\rho _n)-\psi '(r_m)\big ) - r_m\nabla \big (\psi ''(r_m)(\rho _n-r_m)\big ) \end{aligned}$$

and consequently,

$$\begin{aligned}&\int _\Omega \big \{\nabla \big (\psi '(\rho _n)-\psi '(r_m)\big )\cdot (\rho _n u_m) - \nabla \big (\psi ''(r_m)(\rho _n-r_m)\big )\big \}\cdot (r_mu_m)dx \\&\quad = \int _\Omega \nabla p(\rho _n|r_m)\cdot u_m dx = -\int _\Omega p(\rho _n|r_m){\text {div}}u_m dx. \end{aligned}$$

Therefore,

31 $$\begin{aligned}&-\int _\Omega \bigg (\nabla p(\rho _n) - \frac{\rho _n}{r_m}\nabla p(r_m)\bigg )\cdot (v_n-u_m)dx = -\frac{d}{dt}\int _\Omega \psi (\rho _n|r_m)dx \nonumber \\&\quad - \int _\Omega p(\rho _n|r_m){\text {div}}u_m dx + \varepsilon \int _\Omega \big (\psi '(\rho _n)-\psi '(r_m)\big )\Delta \rho _n dx \nonumber \\&\quad - \int _\Omega \psi ''(r_m)(\rho _n-r_m)g_m dx. \end{aligned}$$

*Step 2b: Reformulation of the chemotaxis term.* We reformulate the sixth term on the right-hand side of (29) by integrating by parts and using the mass balances (12) and (26):

32 $$\begin{aligned}&\int _\Omega \rho _n\nabla (c_n-z_m)\cdot (v_n-u_m)dx = -\int _\Omega (c_n-z_m){\text {div}}\big (\rho _n(v_n-u_m)\big )dx \nonumber \\&\quad = \int _\Omega (c_n-z_m){\text {div}}\big (-\rho _nv_n + (\rho _n-r_m)u_m + r_mu_m\big )dx \nonumber \\&\quad = \int _\Omega (c_n-z_m)\big (\partial _t(\rho _n-r_m) + {\text {div}}((\rho _n-r_m)u_m) - \varepsilon \Delta \rho _n + g_m\big )dx \nonumber \\&\quad = \frac{d}{dt}\int _\Omega (c_n-z_m)(\rho _n-r_m)dx - \int _\Omega (\rho _n-r_m)\partial _t(c_n-z_m)dx \nonumber \\&\qquad - \int _\Omega \nabla (c_n-z_m)\cdot ((\rho _n-r_m)u_m)dx - \int _\Omega (c_n-z_m)(\varepsilon \Delta \rho _n-g_m) dx. \end{aligned}$$

In view of the second equation in (26), we have

$$\begin{aligned} \rho _n-r_m = \partial _t(c_n-z_m) - \Delta (c_n-z_m) + (c_n-z_m) + h_m. \end{aligned}$$

We insert this expression into the second term on the right-hand side of (32):

33 $$\begin{aligned}&\int _\Omega \rho _n\nabla (c_n-z_m)\cdot (v_n-u_m)dx = \frac{d}{dt}\int _\Omega (c_n-z_m)(\rho _n-r_m)dx - \int _\Omega |\partial _t(c_n-z_m)|^2 dx \nonumber \\&\quad - \frac{1}{2}\frac{d}{dt}\int _\Omega \bigg (|\nabla (c_n-z_m)|^2 + (c_n-z_m)^2\bigg )dx - \int _\Omega h_m\partial _t(c_n-z_m)dx \nonumber \\&\quad - \int _\Omega \nabla (c_n-z_m)\cdot ((\rho _n-r_m)u_m)dx - \int _\Omega (c_n-z_m)(\varepsilon \Delta \rho _n-g_m) dx. \end{aligned}$$

*Step 2c: Reformulation of the artificial pressure term.* We rewrite the second term on the right-hand side of (29) by integrating by parts and using the mass balance equation (12):

34 $$\begin{aligned}&-\delta \int _\Omega \nabla \rho _n^\beta \cdot (v_n-u_m)dx = -\beta \delta \int _\Omega \rho _n^{\beta -2}\nabla \rho _n\cdot (\rho _n v_n)dx - \delta \int _\Omega \rho _n^\beta {\text {div}}u_m dx \nonumber \\&\quad = \frac{\beta \delta }{\beta -1}\int _\Omega \rho _n^{\beta -1}{\text {div}}(\rho _n v_n)dx - \delta \int _\Omega \rho _n^\beta {\text {div}}u_m dx \nonumber \\&\quad = \frac{\beta \delta }{\beta -1}\int _\Omega \rho _n^{\beta -1}(\varepsilon \Delta \rho _n-\partial _t\rho _n)dx - \delta \int _\Omega \rho _n^\beta {\text {div}}u_m dx \nonumber \\&\quad = -\frac{\delta }{\beta -1}\frac{d}{dt}\int _\Omega \rho _n^\beta dx - \beta \delta \int _\Omega \rho _n^{\beta -2}|\nabla \rho _n|^2 dx - \delta \int _\Omega \rho _n^\beta {\text {div}}u_m dx. \end{aligned}$$

*Step 2d: Collecting the reformulations.* We include the reformulations (31), (33), and (34) into (29) to find that

35 $$\begin{aligned}&\frac{d}{dt}\int _\Omega \bigg \{\frac{1}{2}\rho _n|v_n-u_m|^2 + \psi (\rho _n|r_m) + \frac{1}{2}\bigg (|\nabla (c_n-z_m)|^2 + (c_n-z_m)^2\bigg ) \nonumber \\&\qquad - (c_n-z_m)(\rho _n-r_m) + \frac{\delta }{\beta -1}\rho _n^\beta \bigg \}dx + \int _\Omega |\partial _t(c_n-z_m)|^2dx \nonumber \\&\qquad + \beta \delta 
\int _\Omega \rho _n^{\beta -2}|\nabla \rho _n|^2 dx + \int _\Omega \big (\mu |\nabla (v_n-u_m)|^2 + (\lambda +\mu )|{\text {div}}(v_n-u_m)|^2\big )dx \nonumber \\&\quad = -\int _\Omega p(\rho _n|r_m){\text {div}}u_m dx + \varepsilon \int _\Omega \big (\psi '(\rho _n)-\psi '(r_m)\big )\Delta \rho _n dx \nonumber \\&\qquad - \int _\Omega \psi ''(r_m)(\rho _n-r_m)g_m dx - \int _\Omega h_m\partial _t(c_n-z_m)dx - \delta \int _\Omega \rho _n^\beta {\text {div}}u_m dx \nonumber \\&\qquad - \int _\Omega \nabla (c_n-z_m)\cdot ((\rho _n-r_m)u_m)dx - \int _\Omega (c_n-z_m)(\varepsilon \Delta \rho _n-g_m) dx \nonumber \\&\qquad + \varepsilon \int _\Omega \nabla \rho _n\cdot \nabla u_m\cdot (v_n-u_m)dx - \int _\Omega \rho _n(v_n-u_m)\otimes (v_n-u_m):\nabla u_m dx \nonumber \\&\qquad - \frac{1}{\zeta }\int _\Omega \rho _n|v_n-u_m|^2 dx - \int _\Omega \rho _nf_m\cdot (v_n-u_m)dx \nonumber \\&\qquad - \int _\Omega \frac{\rho _n-r_m}{r_m}\big (\mu \Delta u_m + (\lambda +\mu )\nabla {\text {div}}u_m\big )\cdot (v_n-u_m)dx. \end{aligned}$$

*Step 3: Limit* $(n,m)\rightarrow \infty$ *and* $(\delta ,\varepsilon )\rightarrow 0$. As mentioned in [9, Section 3.3], the limit in the approximate relative energy inequality (35) follows step by step the existence proof in [5, Chapter 7] or [16, Chapter 7]. In particular, we perform first the limit $n\rightarrow \infty$ in the Faedo–Galerkin approximation $(\rho _n,v_n,c_n)\rightarrow (\rho _{\varepsilon ,\delta },v_{\varepsilon ,\delta }, c_{\varepsilon ,\delta })$. Then the functions $(r_m,u_m,z_m)$ are replaced by smooth functions (*r*, *u*, *z*) using a density argument. Third, we pass to the limit $(\rho _{\varepsilon ,\delta },v_{\varepsilon ,\delta },c_{\varepsilon ,\delta })\rightarrow (\rho _\delta ,v_\delta ,c_\delta )$ as $\varepsilon \rightarrow 0$ and $(\rho _\delta ,v_\delta ,c_\delta )\rightarrow (\rho ,v,c)$ as $\delta \rightarrow 0$.

In view of the bounds (20), we can pass to the limit $n\rightarrow \infty$ and $(\delta ,\varepsilon )\rightarrow 0$ in (35). We assume that $(r_m,u_m,z_m)$ converges to (*r*, *u*, *z*) as $m\rightarrow \infty$ in such a way that the limit $m\rightarrow \infty$ in (35) is possible. Then some integrals in (35) disappear and we end up with

$$\begin{aligned}&\frac{d}{dt}\int _\Omega \bigg (\psi (\rho |r) + \frac{1}{2}\rho |v-u|^2 + \frac{1}{2}\big (|\nabla (c-z)|^2 + (c-z)^2\big ) - (\rho -r)(c-z)\bigg )dx \\&\qquad + \int _\Omega \big (\mu |\nabla (v-u)|^2 + (\lambda +\mu )|{\text {div}}(v-u)|^2\big )dx + \int _\Omega |\partial _t(c-z)|^2dx \\&\quad = -\int _\Omega p(\rho |r){\text {div}}u dx - \int _\Omega \psi ''(r)(\rho -r)g dx - \int _\Omega h\partial _t(c-z)dx \\&\qquad - \int _\Omega \nabla (c-z)\cdot ((\rho -r)u)dx + \int _\Omega (c-z)g dx \\&\qquad - \int _\Omega \rho (v-u)\otimes (v-u):\nabla u dx - \frac{1}{\zeta }\int _\Omega \rho |v-u|^2 dx \\&\qquad - \int _\Omega \bigg (\frac{\rho -r}{r}\big (\mu \Delta u + (\lambda +\mu )\nabla {\text {div}}u\big ) + \rho f\bigg )\cdot (v-u)dx. \end{aligned}$$

This shows (10) and finishes the proof. $\square$

## Weak–strong Uniqueness

We split the proof in several steps.

*Step 1: Relative energy inequality.* We claim that (10) holds for finite energy weak solutions $(\rho ,v,c)$ and $({\bar{\rho }},{{\bar{v}}},{{\bar{c}}})$, where $({\bar{\rho }},{{\bar{v}}})$ satisfies the regularity (9). According to [9, Section 4], using a density argument, the relative energy inequality (10) still holds for functions (*r*, *u*) satisfying the following regularity conditions:

36 $$\begin{aligned} \begin{aligned}&r\in C_{\textrm{weak}}^0([0,T];L^\gamma (\Omega )), \quad u\in C^0_{\textrm{weak}}([0,T];L^{2\gamma /(\gamma -1)}(\Omega ;{{\mathbb {R}}}^3)), \\&|\nabla u|\in L^1(0,T;L^\infty (\Omega ))\cap L^2(\Omega \times (0,T)), \quad u=0 \text{ on } \partial \Omega , \\&\partial _t u\in L^1(0,T;L^{2\gamma /(\gamma -1)}(\Omega ;{{\mathbb {R}}}^3))\cap L^2(0,T;L^{6\gamma /(5\gamma -6)}(\Omega ;{{\mathbb {R}}}^3)), \\&|\nabla ^2 u|\in L^1(0,T;L^{2\gamma /(2\gamma +1)}(\Omega ))\cap L^2(0,T;L^{6/5}(\Omega )). \end{aligned} \end{aligned}$$

Moreover, *r* needs to be bounded away from zero and we require $\nabla \psi '(r),\partial _t\psi '(r)\in L^1(0,T;$ $L^{2\gamma /(\gamma -1)}(\Omega ))$. An inspection of (10) reveals that *z* should satisfy

37 $$\begin{aligned} \begin{aligned}&z\in C_{\textrm{weak}}^0([0,T];H^1(\Omega ))\cap H^1(0,T;L^2(\Omega )),\quad \Delta z\in L^2(0,T;L^2(\Omega )), \\&|\nabla z|\in L^1(0,T;L^{2\gamma /(2\gamma -1)}(\Omega ))\cap L^2(0,T;L^{6\gamma /(5\gamma -6)}(\Omega )). \end{aligned} \end{aligned}$$

It follows from [7, Theorem 2.4] that (10) still holds if $(\rho ,v,c)$ is a finite energy weak solution.

### Lemma 8

Let $({\bar{\rho }},{{\bar{v}}},{{\bar{c}}})$ be a finite energy weak solution in the sense of Definition 1 satisfying the additional regularity (9). Furthermore, let ${\bar{c}}^0\in W^{2-2/\gamma ,\gamma }(\Omega )$ and ${\bar{c}}^0\ge 0$ in $\Omega$. Then $({\bar{\rho }},{{\bar{v}}},{{\bar{c}}})$ fulfills the regularity conditions (36)–(37).

### Proof

Regularity (36) follows as in [9, Section 4] from Sobolev embeddings. Theorem 10 in the Appendix shows that (37) is satisfied. $\square$

The previous lemma shows that we can take $(r,u,z)=({\bar{\rho }},{\bar{v}},{\bar{c}})$ in (10). Then the remainder $R(\rho ,v,c|{\bar{\rho }},{\bar{v}},{\bar{c}})$ in Lemma 7 simplies, since $f=0$ and $g=h=0$, and we find that

38 $$\begin{aligned}&\int _0^t R(\rho ,v,c|{\bar{\rho }},{\bar{v}},{\bar{c}})ds = J_1+\cdots + J_5, \quad \text{ where } \nonumber \\&J_1 = -\int _0^t\int _\Omega p(\rho |{\bar{\rho }}){\text {div}}{\bar{v}}dxds, \nonumber \\&J_2 = -\int _0^t\int _\Omega \nabla (c-{\bar{c}})\cdot ((\rho -{\bar{\rho }}){\bar{v}})dxds, \nonumber \\&J_3 = -\int _0^t\int _\Omega \rho (v-{{\bar{v}}})\otimes (v-{{\bar{v}}}):\nabla {{\bar{v}}} dxds, \nonumber \\&J_4 = -\frac{1}{\zeta }\int _0^t\int _\Omega {\bar{\rho }}|v-{{\bar{v}}}|^2 dxds, \nonumber \\&J_5 = -\int _0^t\int _\Omega \frac{\rho -{\bar{\rho }}}{{\bar{\rho }}}\big (\mu \Delta {{\bar{v}}} + (\lambda +\mu )\nabla {\text {div}}{{\bar{v}}}\big )\cdot (v-{{\bar{v}}})dxds. \end{aligned}$$

*Step 2: Estimation of* $J_i$. The terms $J_i$ can be estimated as in [9, Section 4.1] except the new term $J_2$. Indeed, since $p(\rho )=(\gamma -1)\psi (\rho )$, we have $p(\rho |{\bar{\rho }})=(\gamma -1)\psi (\rho |{\bar{\rho }})$, showing that

$$\begin{aligned} J_1 \le C\int _0^t\int _\Omega \psi (\rho |{\bar{\rho }})dxds, \end{aligned}$$

and Hölder’s inequality gives

$$\begin{aligned} J_3 \le C\int _0^t\int _\Omega \rho |v-{{\bar{v}}}|^2 dxds, \end{aligned}$$

where $C>0$ depends on the $L^\infty (\Omega \times (0,T))$ norm of $\nabla {{\bar{v}}}$. The term $J_4$ is nonpositive and can be neglected. Formulas (4.13)–(4.14) in [9] lead to

$$\begin{aligned} J_5 \le \xi \int _0^t\Vert v-{{\bar{v}}}\Vert _{H^1(\Omega )}^2 ds + C(\xi )\int _0^t\int _\Omega \psi (\rho |{\bar{\rho }})dxds, \end{aligned}$$

where $\xi >0$ is arbitrary and $C(\xi )>0$ depends on $\xi$ as well as $\Vert {{\bar{v}}}\Vert _{L^\infty (0,t;W^{2,3}(\Omega ))}$ and $\Vert \nabla ^2{{\bar{v}}}\Vert _{L^\infty (0,t;L^q(\Omega ))}$. At this point, we need the condition $q>3$.

To estimate the term $J_2$, which is not contained in [9], we use equation (3) for *c* and integrate by parts:

$$\begin{aligned} J_2&= -\int _0^t\int _\Omega \nabla (c-{{\bar{c}}})\cdot {{\bar{v}}}\big (\partial _t(c-{{\bar{c}}}) - \Delta (c-{{\bar{c}}}) + (c-{{\bar{c}}})\big )dx \\&= -\int _0^t\int _\Omega \partial _t(c-{{\bar{c}}})\nabla (c-{{\bar{c}}})\cdot {{\bar{v}}} dxds - \frac{1}{2}\int _0^t\int _\Omega \nabla [(c-{{\bar{c}}})^2]\cdot {{\bar{v}}} dxds \\&\quad + \int _0^t\int _\Omega \bigg ({\text {div}}\big (\nabla (c-{{\bar{c}}})\otimes \nabla (c-{{\bar{c}}})\big ) - \frac{1}{2}\nabla |\nabla (c-{{\bar{c}}})|^2\bigg )\cdot {{\bar{v}}}dxds \\&= -\int _0^t\int _\Omega \partial _t(c-{{\bar{c}}})\nabla (c-{{\bar{c}}})\cdot {{\bar{v}}} dxds + \frac{1}{2}\int _0^t\int _\Omega (c-{{\bar{c}}})^2{\text {div}}{{\bar{v}}} dxds \\&\quad - \int _0^t\int _\Omega \bigg (\nabla (c-{{\bar{c}}})\otimes (c-{{\bar{c}}}):\nabla {{\bar{v}}} - \frac{1}{2}|\nabla (c-{{\bar{c}}})|^2{\text {div}}{{\bar{v}}}\bigg )dxds. \end{aligned}$$

Then, by Young’s inequality,

$$\begin{aligned} J_2 \le \frac{1}{2}\int _0^t\int _\Omega |\partial _s(c-{{\bar{c}}})|^2 dxds + C\int _0^t\int _\Omega \big (|\nabla (c-{{\bar{c}}})|^2 + (c-{{\bar{c}}})^2\big )dxds, \end{aligned}$$

where $C>0$ depends on $\Vert {{\bar{v}}}\Vert _{L^\infty (0,T;W^{1,\infty }(\Omega ))}$. Summarizing, it follows from (38) that

$$\begin{aligned}&\int _0^t R(\rho ,v,c|{\bar{\rho }},{{\bar{v}}},{{\bar{c}}})ds \le \frac{1}{2}\int _0^t\int _\Omega |\partial _s(c-{{\bar{c}}})|^2 dxds + \xi \int _0^t\Vert v-{{\bar{v}}}\Vert _{H^1(\Omega )}^2 ds \\&\quad + C\int _0^t\int _\Omega \big (\psi (\rho |{\bar{\rho }}) + \rho |v-{{\bar{v}}}|^2 + |\nabla (c-{{\bar{c}}})|^2 + (c-{{\bar{c}}})^2\big )dxds. \end{aligned}$$

The first term on the right-hand side can be absorbed by the last term on the left-hand side of (10). The second term on the left-hand side of (10) can be bounded from below by Korn’s inequality [15, Lemma 2] according to

$$\begin{aligned} \int _\Omega \big (\mu |\nabla (v-{{\bar{v}}})|^2 + (\lambda +\mu )|{\text {div}}(v-{{\bar{v}}})|^2\big )dx \ge C_K\Vert v-{{\bar{v}}}\Vert _{H^1(\Omega )}^2, \end{aligned}$$

since $v={{\bar{v}}}=0$ on $\partial \Omega$. Therefore, choosing $0<\xi <C_K$, (10) yields

39 $$\begin{aligned}&E((\rho ,v,c)(t)|({\bar{\rho }},{{\bar{v}}},{{\bar{c}}})(t)) + \frac{1}{2}\int _0^t\int _\Omega |\partial _s(c-{{\bar{c}}})|^2 dxds + (C_K-\xi )\int _0^t\Vert v-{{\bar{v}}}\Vert _{H^1(\Omega )}^2 ds \nonumber \\&\quad \le E(\rho _0,v_0,c_0|{\bar{\rho }}_0,{{\bar{v}}}_0,{{\bar{c}}}_0) \nonumber \\&\qquad + C\int _0^t\int _\Omega \big (\psi (\rho |{\bar{\rho }}) + \rho |v-{{\bar{v}}}|^2 + |\nabla (c-{{\bar{c}}})|^2 + (c-{{\bar{c}}})^2\big )dxds \nonumber \\&\quad \le E(\rho _0,v_0,c_0|{\bar{\rho }}_0,{{\bar{v}}}_0,{{\bar{c}}}_0) + C\int _0^t H(\rho ,v,c|{\bar{\rho }},{\bar{v}},{\bar{c}})ds. \end{aligned}$$

*Step 3: Estimation of* $\int _\Omega (\rho -{\bar{\rho }})(c-{{\bar{c}}})dx$. We use Lemma 9 in Appendix 4 with $m=2$ and arbitrary $\kappa _1,\xi >0$ on the set $\{\rho \le R\}$ for some $R>0$:

40 $$\begin{aligned} \int _{\{\rho \le R\}}(\rho -{\bar{\rho }})(c-{{\bar{c}}})dx&\le \kappa _1\Vert \rho -{\bar{\rho }}\Vert _{L^2(\Omega \cap \{\rho \le R\})}^2 + \xi \Vert \nabla (c-{{\bar{c}}})\Vert _{L^2(\Omega )}^2 \nonumber \\&\quad + C_1(\kappa _1,\xi )\Vert c-{{\bar{c}}}\Vert _{L^1(\Omega )}^{C_2(2)}, \end{aligned}$$

as well as with $m=\gamma$ (which requires $\gamma >8/5$) and arbitrary $\kappa _2>0$ on the set $\{\rho >R\}$:

41 $$\begin{aligned} \int _{\{\rho>R\}}(\rho -{\bar{\rho }})(c-{{\bar{c}}})dx&\le \kappa _2\Vert \rho -{\bar{\rho }}\Vert _{L^\gamma (\Omega \cap \{\rho >R\})}^\gamma + \xi \Vert \nabla (c-{{\bar{c}}})\Vert _{L^2(\Omega )}^2 \nonumber \\&\quad + C_1(\kappa _2,\xi )\Vert c-{{\bar{c}}}\Vert _{L^1(\Omega )}^{C_2(\gamma )}. \end{aligned}$$

According to [13, Lemma 2.4], there exist constants $C_3,C_4,c_p,C_p>0$ such that

$$\begin{aligned} \psi (\rho |{\bar{\rho }}) \ge {\left\{ \begin{array}{ll} C_3|\rho -{\bar{\rho }}|^2 &{}\quad \text{ if } 0\le \rho \le R, \\ C_4|\rho -{\bar{\rho }}|^\gamma &{}\quad \text{ if } \rho >R, \end{array}\right. } \end{aligned}$$

as long as $c_p\le {\bar{\rho }}\le C_p$. Thus, we can replace the first term on the right-hand sides of (40) and (41), respectively, by $\kappa _1 C_3^{-1}\int _\Omega \psi (\rho |{\bar{\rho }})dx$ and $\kappa _2 C_4^{-1} \int _\Omega \psi (\rho |{\bar{\rho }})dx$, and summing these inequalities, we obtain

42 $$\begin{aligned} \int _\Omega (\rho -{\bar{\rho }})(c-{{\bar{c}}})dx&\le \bigg (\frac{\kappa _1}{C_3}+\frac{\kappa _2}{C_4}\bigg )\int _\Omega \psi (\rho |{\bar{\rho }})dx + 2\xi \Vert \nabla (c-{{\bar{c}}})\Vert _{L^2(\Omega )}^2 \nonumber \\&\quad + C_1(\kappa _1,\xi )\Vert c-{{\bar{c}}}\Vert _{L^1(\Omega )}^{C_2(2)} + C_1(\kappa _2,\xi )\Vert c-{{\bar{c}}}\Vert _{L^1(\Omega )}^{C_2(\gamma )}. \end{aligned}$$

We wish to estimate the last two norms in terms of the initial data. To this end, we integrate (3) and use the mass conservation $\Vert \rho (t)\Vert _{L^1(\Omega )}=\Vert \rho ^0\Vert _{L^1(\Omega )}$ and $\Vert {\bar{\rho }}(t)\Vert _{L^1(\Omega )}=\Vert {\bar{\rho }}^0\Vert _{L^1(\Omega )}$:

$$\begin{aligned} \frac{d}{dt}\int _\Omega (c-{{\bar{c}}})(t)dx&= -\int _\Omega (c-{{\bar{c}}})dx + \int _\Omega (\rho -{\bar{\rho }})dx = -\int _\Omega (c-{{\bar{c}}})dx + \int _\Omega (\rho ^0-{\bar{\rho }}^0)dx. \end{aligned}$$

Gronwall’s lemma yields

$$\begin{aligned} \int _\Omega (c-{{\bar{c}}})(t)dx \le C\int _\Omega (c^0-{{\bar{c}}}^0)dx + C\int _\Omega (\rho ^0-{\bar{\rho }}^0)dx. \end{aligned}$$

The same argument with ${{\bar{c}}}-c$ then shows that

$$\begin{aligned} \Vert (c-{{\bar{c}}})(t)\Vert _{L^1(\Omega )} \le C\big (\Vert c^0-{{\bar{c}}}^0\Vert _{L^1(\Omega )} + \Vert \rho ^0-{\bar{\rho }}^0\Vert _{L^1(\Omega )}\big ). \end{aligned}$$

Hence, choosing $\kappa _1=C_3/4$, $\kappa _2=C_4/4$, and $\xi =1/8$, we deduce from (42) that

$$\begin{aligned} \int _\Omega (\rho -{\bar{\rho }})(c-{{\bar{c}}})dx&\le \frac{1}{2}\int _\Omega \bigg (\psi (\rho |{\bar{\rho }}) + \frac{1}{2}|\nabla (c-{{\bar{c}}})|^2\bigg )dx \\&\quad + C_1(\kappa _1,\xi )\big (\Vert c^0-{{\bar{c}}}^0\Vert _{L^1(\Omega )} + \Vert \rho ^0-{\bar{\rho }}^0\Vert _{L^1(\Omega )}\big )^{C_2(2)} \\&\quad + C_1(\kappa _2,\xi )\big (\Vert c^0-{{\bar{c}}}^0\Vert _{L^1(\Omega )} + \Vert \rho ^0-{\bar{\rho }}^0\Vert _{L^1(\Omega )}\big )^{C_2(\gamma )}. \end{aligned}$$

The last two terms are bounded from above by

$$\begin{aligned} C\big (\Vert c^0-{{\bar{c}}}^0\Vert _{L^1(\Omega )} + \Vert \rho ^0-{\bar{\rho }}^0\Vert _{L^1(\Omega )}\big )^{C_5}, \end{aligned}$$

where $C_5$ equals $C_2(2)$ or $C_2(\gamma )$ depending on whether $\Vert c^0-{{\bar{c}}}^0\Vert _{L^1(\Omega )}+\Vert \rho ^0-{\bar{\rho }}^0\Vert _{L^1(\Omega )}$ is smaller or larger than one. We conclude that

43 $$\begin{aligned} \int _\Omega (\rho -{\bar{\rho }})(c-{{\bar{c}}})dx&\le \frac{1}{2}\int _\Omega \bigg (\psi (\rho |{\bar{\rho }}) + \frac{1}{2}|\nabla (c-{{\bar{c}}})|^2\bigg )dx \nonumber \\&\quad + C\big (\Vert c^0-{{\bar{c}}}^0\Vert _{L^1(\Omega )} + \Vert \rho ^0-{\bar{\rho }}^0\Vert _{L^1(\Omega )}\big )^{C_5} \nonumber \\&\le \frac{1}{2} H(\rho ,v,c|{\bar{\rho }},{{\bar{v}}},{{\bar{c}}}) + C\big (\Vert c^0-{{\bar{c}}}^0\Vert _{L^1(\Omega )} + \Vert \rho ^0-{\bar{\rho }}^0\Vert _{L^1(\Omega )}\big )^{C_5}. \end{aligned}$$

*Step 4: End of the proof.* By (43), the relative energy is bounded from below by

$$\begin{aligned} E(\rho ,v,c|{\bar{\rho }},{{\bar{v}}},{{\bar{c}}})&\ge H(\rho ,v,c|{\bar{\rho }},{{\bar{v}}},{{\bar{c}}}) - \int _\Omega (\rho -{\bar{\rho }})(c-{{\bar{c}}})dx \\&\ge \frac{1}{2}H(\rho ,v,c|{\bar{\rho }},{{\bar{v}}},{{\bar{c}}}) - C\big (\Vert c^0-{{\bar{c}}}^0\Vert _{L^1(\Omega )} + \Vert \rho ^0-{\bar{\rho }}^0\Vert _{L^1(\Omega )}\big )^{C_5}. \end{aligned}$$

We insert this estimate into (39):

$$\begin{aligned}&\frac{1}{2} H((\rho ,v,c)(t)|({\bar{\rho }},{{\bar{v}}},{{\bar{c}}})(t)) + \frac{1}{2}\int _0^t\int _\Omega |\partial _s(c-{{\bar{c}}})|^2 dxds + C\int _0^t\Vert v-{{\bar{v}}}\Vert _{H^1(\Omega )}^2 ds \\&\quad \le E(\rho ^0,v^0,c^0|{\bar{\rho }}^0,{{\bar{v}}}^0,{{\bar{c}}}^0) + C\int _0^t H(\rho ,v,c|{\bar{\rho }},{{\bar{v}}},{{\bar{c}}})ds \\&\qquad + C\big (\Vert c^0-{{\bar{c}}}^0\Vert _{L^1(\Omega )} + \Vert \rho ^0-{\bar{\rho }}^0\Vert _{L^1(\Omega )}\big )^{C_5}. \end{aligned}$$

An application of Gronwall’s lemma gives

$$\begin{aligned} H((\rho ,v,c)(t)|({\bar{\rho }},{{\bar{v}}},{{\bar{c}}})(t))&\le C e^{Ct}\big \{E(\rho ^0,v^0,c^0|{\bar{\rho }}^0,{{\bar{v}}}^0,{{\bar{c}}}^0) \\&\quad + \big (\Vert c^0-{{\bar{c}}}^0\Vert _{L^1(\Omega )} + \Vert \rho ^0-{\bar{\rho }}^0\Vert _{L^1(\Omega )}\big )^{C_5}\big \}, 
\end{aligned}$$

and the choice $\rho ^0={\bar{\rho }}^0$, $v^0={{\bar{v}}}^0$, $c^0={{\bar{c}}}^0$ ends the proof.

## Appendix A: Auxiliary Results

### Lemma 9

Let $\Omega \subset {{\mathbb {R}}}^d$ be a bounded domain with $d\in \{2,3\}$ and let $m>2(d+1)/(d+2)$. Furthermore, let $\kappa ,\xi >0$. Then there exist constants $C_1(\kappa ,\xi )>0$ and $C_2(m)>0$ such that for all $\rho \in L^m(\Omega )$, $c\in H^1(\Omega )$,

$$\begin{aligned} \int _\Omega \rho cdx \le \kappa \Vert \rho \Vert _{L^m(\Omega )}^m + \xi \Vert \nabla c\Vert _{L^2(\Omega )}^2 + C_1(\kappa ,\xi )\Vert c\Vert _{L^1(\Omega )}^{C_2(m)}. \end{aligned}$$

### Proof

The proof of the lemma is contained in [18, Appendix B] for solutions to the degenerate Keller–Segel equations. For clarity, we present the proof for general functions $\rho$ and *c*. We conclude from the interpolation inequality for Lebesgue spaces and Young’s inequality that for any $\kappa >0$,

$$\begin{aligned} \Vert \rho c\Vert _{L^1(\Omega )} \le \Vert \rho \Vert _{L^m(\Omega )}\Vert c\Vert _{L^{m/(m-1)}(\Omega )} \le \kappa \Vert \rho \Vert _{L^m(\Omega )}^m + C(\kappa )\Vert c\Vert _{L^{m/(m-1)}(\Omega )}^{m/(m-1)}. \end{aligned}$$

We estimate the second term on the right-hand side by applying the Gagliardo–Nirenberg inequality with $\theta =2d/(m(d+2))$:

$$\begin{aligned} \Vert c\Vert _{L^{m/(m-1)}(\Omega )} \le C\Vert \nabla c\Vert _{L^2(\Omega )}^\theta \Vert c\Vert _{L^1(\Omega )}^{1-\theta } + C\Vert c\Vert _{L^1(\Omega )}. \end{aligned}$$

Then, by Minkowski’s and Young’s inequality, for any $\varepsilon >0$,

$$\begin{aligned} \Vert \rho c\Vert _{L^1(\Omega )}&\le \kappa \Vert \rho \Vert _{L^m(\Omega )}^m + C(\kappa ,m)\big (\Vert \nabla c\Vert _{L^2(\Omega )}^{m\theta /(m-1)} \Vert c\Vert _{L^1(\Omega )}^{m(1-\theta )/(m-1)} + \Vert c\Vert _{L^1(\Omega )}^{m/(m-1)}\big ) \\&\le \kappa \Vert \rho \Vert _{L^m(\Omega )}^m + C(\kappa ,m)\varepsilon \Vert \nabla c\Vert _{L^2(\Omega )}^2 \\&\quad + C(\kappa ,m,\varepsilon )\big (\Vert c\Vert _{L^1(\Omega )}^{2m(1-\theta )/(2(m-1)-m\theta )} + \Vert c\Vert _{L^1(\Omega )}^{m/(m-1)}\big ), \end{aligned}$$

which is possible since $m\theta /(m-1)<2$ is equivalent to $m>2(d+1)/(d+2)$. The lemma follows after choosing $\varepsilon =\xi /C(\kappa ,m)$, $C_1(\kappa ,\xi )=C(\kappa ,m,\varepsilon )$, and $C_2(m)=\max \{m/(m-1),2m(1-\theta )/(2(m-1)-m\theta )\}$.

$$\square $$

The following result concerns the maximal regularity of the solution to

44 $$\begin{aligned}&\partial _t u - \Delta u + u = f\quad \text{ in } \Omega ,\ t>0, \end{aligned}$$

45 $$\begin{aligned}&\nabla u\cdot \nu =0 \text{ on } \partial \Omega ,\ t>0, \quad u(\cdot ,0)=u^0 \text{ in } \Omega , \end{aligned}$$

where $\Omega \subset {{\mathbb {R}}}^d$ ($d\ge 1$) is a bounded domain with $C^3$ boundary. We recall that $W^{2,-2/p,q}_\nu (\Omega )$ is the completion of the space of functions $w\in C^\infty ({\overline{\Omega }})$ satisfying $\nabla w\cdot \nu =0$ on $\partial \Omega$ in the norm of $W^{2-2/p,q}(\Omega )$. The theorem is a special case of [6, Theorem 10.22] or [16, Lemma 7.37].

### Theorem 10

*(Maximal regularity)*. Let $1<p,q<\infty$, $f\in L^p(0,T;L^q(\Omega ))$, and let $u^0\in W^{2-2/p,q}_\nu (\Omega )$. Then there exists a unique solution *u* to (44)–(45) satisfying

$$\begin{aligned} u\in L^p(0,T;W^{2,q}(\Omega ))\cap W^{1,p}(0,T;L^q(\Omega ))\cap C^0([0,T];W^{2-2/p,q}(\Omega )), \end{aligned}$$

and there exists a constant $C>0$ such that

$$\begin{aligned}&\Vert u\Vert _{L^\infty (0,T;W^{2-2/p,q}(\Omega ))} + \Vert u\Vert _{L^p(0,T;W^{2,q}(\Omega ))} + \Vert \partial _t u\Vert _{L^p(0,T;L^q(\Omega ))} \\&\quad \le C\big (\Vert f\Vert _{L^p(0,T;L^q(\Omega ))} + \Vert u^0\Vert _{W^{2-2/p,q}(\Omega )}\big ). \end{aligned}$$
